# Supplementary material for: Association of Genomic Alterations with the Presence of Serum Monoclonal Proteins in Chronic Lymphocytic Leukemia
Source: Cells. 2024 Nov 7;13(22):1839. doi: 10.3390/cells13221839 (PMC11592641; doi:10.3390/cells13221839)
Supplement: Supplementary file 1 [file cells-13-01839-s001.zip › cells-3244281-supplementary.pdf]

Supplementary Tables

Supplementary Table S1

Baseline clinical and laboratory characteristics according to sample timepoint.

**Characteristic**	**All patients (n=97)**	**Time when sIFE and NGS were performed**			
		**Diagnosis (n=59, 61%)**	**Follow up (n=17, 17%)**	**Before treatment (n=21, 22%)**	**p value**
sIFE	49 (51)	30 (51)	6 (35)	13 (62)	NS
Age in years, median (range)	69 (41-96)	70 (41-94)	69 (57-96)	69 (44-80)	NS
Male sex, n (%)	57 (59)	33 (56)	10 (59)	14 (67)	NS
Diagnosis					
CLL, n (%)	89 (92)	51 (86)	17 (100)	21 (100)	
MBL, n (%)	5 (5)	5 (8)	0	0	NS
SLL, n (%)	3(3)	3 (5)	0	0	
Binet stage C, n (%)	13 (13)	4 (7)	1 (6)	8 (38)	NS
Rai stage III-IV, n (%)	14 (14)	5 (8)	1 (6)	8 (38)	NS
ALC >15x109/L, n (%)	56 (58)	25 (42)	16 (88)	16 (76)	NS
LDH above ULN, n (%) (n=92)	13 (14)	7 (12)	2 (13)	4 (24)	NS
β2-microglobulin above ULN, n (%) (n=91)	52 (57)	38 (64)	7 (44)	7 (41)	NS
Complex karyotype (n=88)	7 (8)	4 (7)	2 (13)	1 (6)	NS
FISH, n (%)					
Normal	15 (16)	10 (18)	1 (7)	4 (19)	
del(13)(q14.3)	37 (40)	23 (40)	6 (43)	8 (38)	
Trisomy 12	13 (14)	5 (9)	4 (29)	4 (19)	NS
del(11)(q22.3)	8 (9)	4 (7)	1 (7)	3 (14)	
del(17)(p13.1)	19 (21)	15 (26)	2 (14)	2 (10)	
Diagnosis period					
2003-2007	3 (3)	0	0	3 (14)	
2008-2012	13 (13)	2 (4)	6 (35)	5 (24)	<0.001
2013-2017	19 (20)	9 (15)	8 (47)	2 (10)	
2018-2023	62 (64)	48 (81)	3 (18)	11 (52)	
NGS and sIFE date					
2003-2007	0	0	0	0	
2008-2012	3 (3)	1 (2)	2 (12)	0	
2013-2017	14 (14)	8 (14)	3 (18)	18 (46)	NS
2018-2023	80 (83)	50 (84)	12 (70)	21 (54)	

ALC, absolute lymphocyte count; NGS, next generation sequencing; NS, not statistically significant; sIFE, serum immunofixation electrophoresis. MBL, CLL-type monoclonal B-cell lymphocytosis; NS, not statistically significant; SLL, small lymphocytic lymphoma; CLL, chronic lymphocytic leukemia; LDH, lactate dehydrogenase; ULN, upper limit of normal.

Supplementary Table S2 Outcomes with BTKi as first line of treatment.

Variable by BTKi	Patients (n=29)	sIFE at the time of NGS Negative (n=15, 51%)	Positive (n=14, 49%)
**Ibrutinib, n (%)**	**20 (70)**	**11 (74)**	**9 (65)**
Dosage, n (%)			
420 mg/24h	18 (90)	10 (92)	8 (88)
280 mg/24h	2 (10)	1 (8)	1 (11)
Duration of treatment, median (range)	2.2y (0.1-6.7y)	2.7y (0.1-6.7y)	1.3y (0.1-5.3y)
Response, n (%)			
CR	7 (35)	5 (45)	2 (22)
PR	12 (60)	5 (45)	7 (78)
PD	1 (5)	1 (9)	0
Toxicity, n (%)			
Arrhythmia	4 (19)	1 (8)	3 (33)
Bleeding	1 (5)	1 (8)	0
Infection	1 (5)	1 (8)	0
Arthralgia	2 (10)	1 (8)	1 (11)
Diarrhea	1 (5)	1 (8)	0
Discontinuation, n (%)			
Progression	3 (14)	1 (8)	2 (22)
Toxicity	5 (24)	3 (25)	2 (22)
Death	3 (14)	1 (8)	2 (22)
Surgery	1 (5)	0	1 (11)
**Acalabrutinib, n (%)**	**5 (17)**	**2 (13)**	**3 (21)**
Dosage, n (%)			
100 mg/12h	4 (80)	2 (100)	2 (67)
100 mg/24h	1 (20)	0	1 (33)
Duration of treatment, median (range)	0.6y (0.5-0.7y)	0.6y (0.5-0.6y)	0.7y (0.6-0.7y)
Response, n (%)			
CR	3 (60)	1 (50)	2 (60)
SD	2 (40)	1 (50)	1 (40)
Toxicity, n (%)			
Infection	1 (20)	0	1 (33)
Fatigue	1 (20)	1 (50)	0
Discontinuation, n (%)			
Progression	1 (20)	1 (50)	0
**Zanubrutinib, n (%)**	**4 (13)**	**2 (13)**	**2 (14)**
Dosage, n (%)			
320 mg/24h	3 (75)	2 (100)	1 (50)
160 mg/24h	1 (25)	0	1 (50)
Duration of treatment, median (range)	0.4y (0.2-0.6y)	0.5y (0.3-0.6y)	0.4y (0.2-0.5y)
Response, n (%)			
CR	1 (25)	1 (50)	0
PR	1 (25)	0	1 (50)
NA	2 (50)	1 (50)	1 (50)
Toxicity, n (%)			
Diarrhea	1 (25)	1 (50)	0
Discontinuation, n (%)	0	0	0

BCL2i, B-cell lymphoma 2 inhibitor; BTKi, Bruton tyrosine kinase inhibitors, CR, complete remission; PD, progressive disease; PR, partial remission; SD, stable disease; NGS, next generation sequencing; sIFE, serum immunofixation electrophoresis.NA, not available; y, year.
